# Feasibility of Training Clinical Officers in Point-of-Care Ultrasound for Pediatric Respiratory Diseases in Aweil, South Sudan

**DOI:** 10.4269/ajtmh.18-0745

**Published:** 2019-07-08

**Authors:** Adi Nadimpalli, James W. Tsung, Ramon Sanchez, Sachita Shah, Evgenia Zelikova, Lisa Umphrey, Northan Hurtado, Alan Gonzalez, Carrie Teicher

**Affiliations:** 1 Médecins Sans Frontières, Aweil, South Sudan;; 2 Department of Emergency Medicine, Icahn School of Medicine at Mount Sinai, New York, New York;; 3 Department of Pediatrics, Icahn School of Medicine at Mount Sinai, New York, New York;; 4 Department of Radiology, University of Michigan School of Medicine, Ann Arbor, Michigan;; 5 Department of Emergency Medicine, University of Washington, Seattle, Washington;; 6 Médecins Sans Frontières, Juba, South Sudan;; 7 Médecins Sans Frontières Medical Department, Sydney, Australia;; 8 Médecins Sans Frontières Medical Department, New York, New York;; 9 Epicentre, New York, New York

## Abstract

Lower respiratory tract infections (LRTIs) are the leading cause of deaths in children < 5 years old worldwide, particularly affecting low-resource settings such as Aweil, South Sudan. In these settings, diagnosis can be difficult because of either lack of access to radiography or clinical algorithms that overtreat children with antibiotics who only have viral LRTIs. Point-of-care ultrasound (POCUS) has been applied to LRTIs, but not by nonphysician clinicians, and with limited data from low-resource settings. Our goal was to examine the feasibility of training the mid-level provider cadre clinical officers (COs) in a Médecins Sans Frontières project in South Sudan to perform a POCUS algorithm to differentiate among causes of LRTI. Six COs underwent POCUS training, and each subsequently performed 60 lung POCUS studies on hospitalized pediatric patients < 5 years old with criteria for pneumonia. Two blinded experts, with a tiebreaker expert adjudicating discordant results, served as a reference standard to calculate test performance characteristics, assessed image quality and CO interpretation. The COs performed 360 studies. Reviewers rated 99.1% of the images acceptable and 86.0% CO interpretations appropriate. The inter-rater agreement (κ) between COs and experts for lung consolidation with air bronchograms was 0.73 (0.63–0.82) and for viral LRTI/bronchiolitis was 0.81 (0.74–0.87). It is feasible to train COs in South Sudan to use a POCUS algorithm to diagnose pneumonia and other pulmonary diseases in children < 5 years old.

## INTRODUCTION

Pneumonia is the single largest infectious cause of death in children worldwide, killing 2,500 children younger than 5 years a day and accounting for 15% of all under-five deaths globally.^1^ In South Sudan, where infant and under-five mortality rates are very high at 75 and 105 deaths per 1,000 live births, respectively, pneumonia contributes to 20% of deaths in children younger than five years.^2^

Accurate diagnosis and proper management of pneumonia can be challenging, especially in low-resource settings where skilled clinicians are limited, and standard imaging may be unavailable.^3^ Thus, many children diagnosed clinically with pneumonia have viral infections only, leading to suboptimal antibiotic stewardship and concern for increasing antibiotic resistance.^4^ There has been significant interest in using portable ultrasound technology in low- and middle-income countries (LMICs), as it requires significantly less infrastructure and training than the current gold standard diagnostic imaging using chest X-rays.^5^ Point-of-care ultrasound (POCUS) is a widely used clinical imaging method for rapid diagnosis, can expedite treatment at the bedside, and is relatively easy to learn.^6–8^ It can be brought to wherever the patient is located and does not emit radiation. A meta-analysis by Pereda et al.^9^ shows POCUS sensitivities and specificities to be greater than 90% for diagnosing pneumonia in children. The work by Reali et al.^10^ in 2014 showed that lung ultrasounds can be at least as effective as chest X-ray in diagnosing pneumonias in pediatric patients. Furthermore, a 2015 study by Chavez et al.^11^ in two resource-limited settings shows that lung POCUS can be taught efficiently to general practitioners because the use of POCUS is based on simple techniques and pattern recognition.

This study evaluates a training program undertaken in Aweil, South Sudan, where significant patient care is provided by clinical officers (COs), mid-level clinicians with 3 years of medical education, using a variant of the task-shifting model.^12,13^ The study aims to demonstrate that in resource-poor contexts with a shortage of medical doctors, it is feasible for nonphysician clinicians, specifically COs, to diagnose respiratory pathologies using a validated POCUS algorithm. Of note, COs are mid-level medical providers in South Sudan, being qualified after 3 years of education. Médecins Sans Frontières (MSF) has been operating a maternal and child health program at the Aweil State Hospital since 2008, in collaboration with the Ministry of Health (MOH), the Government of South Sudan, and State MOHs. The objective of the program is to reduce pediatric and maternal mortality in Aweil town and catchment area of the state hospital through access to free secondary pediatric and gynecology/obstetric care.

## MATERIALS AND METHODS

### Ethics.

All parents or guardians gave written informed consent for eligible patients to participate in the study and were not compensated for participation in the study. The Ethical Committee of the MOH in South Sudan gave approval for publication.

### Study design.

The objective was to determine if ultrasound-naive South Sudanese COs on staff at the MSF project in Aweil could learn an algorithm already described in the research.^14^ This was a feasibility study evaluating the training of six South Sudanese COs’ capacity to diagnose respiratory pathologies by lung ultrasound. The COs underwent a 12-hour field-based training, which included both didactic and practical components using a Philips Lumify linear probe (5–12 mhz) (Bothell, WA) and a Nvidia Shield 2 tablet (Santa Clara, CA). Subsequently, over a 6-week period, each performed and analyzed 60 lung ultrasound studies on a convenience sample of admitted children less than 5 years old, which were then graded by expert reviewers. Patients were eligible for ultrasound study by a CO if they had clinical signs of lower respiratory tract infection (LRTI) in the MSF pediatric services between January and March of 2017. All the children who received an ultrasound study had either 1) a clinical diagnosis of LRTI/pneumonia or 2) fit the clinical criteria for LRTI/pneumonia (cough/difficulty breathing with either age-appropriate tachypnea or intercostal retractions) while having another diagnosis (e.g., malaria). These eligible children were enrolled into the study from the general inpatient department, inpatient therapeutic feeding department, intensive care unit, and emergency department. The guardians of all eligible children were asked for written informed consent to participate in this CO training and have their images saved. We included only patients whose parent or guardians gave written informed consent to be enrolled in the study. No clinical decision-making was altered during this training, as participating COs were not responsible for care of these patients. Patients were cared for by staff physicians, and staff COs were not involved in the study.

### Point-of-care ultrasound training for COs.

The training for the COs consisted of two 6-hour training sessions per day, over two consecutive days. Each session included a 1-hour didactic session, followed by a 2-hour bedside teaching session, and concluded with a 3-hour window to practice using the device. Trainings were conducted by a pediatrician who completed a POCUS fellowship. Before the training, all COs completed a questionnaire to assess their background knowledge in ultrasound.

**Figure 1.**
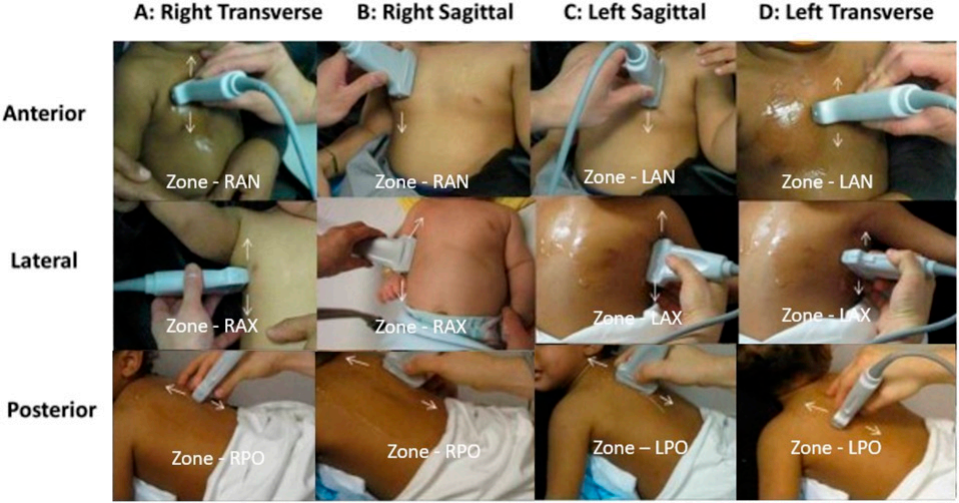
Six zone, 12 view lung ultrasound scan protocol.^14^ This figure appears in color at www.ajtmh.org.

**Figure 2.**
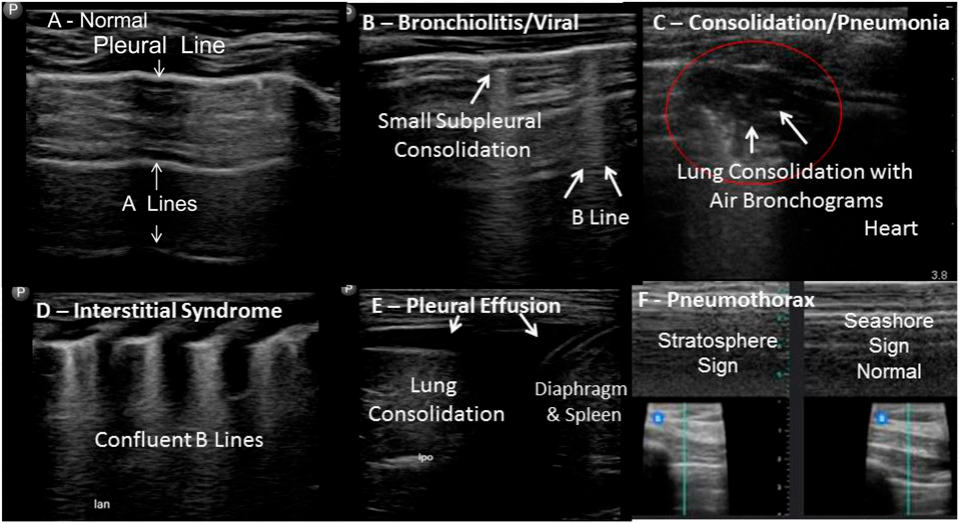
Examples of ultrasound findings. Interstitial syndrome refers to acute respiratory distress syndrome or severe viral LRTI, less commonly pulmonary edema.^15^

The COs used a six-zone technique for lung ultrasound (Figure 1) described by Tsung et al.^14^ In each zone, the CO acquired both longitudinal and transverse views, equaling 12 total views. After image acquisition, the COs analyzed their images according to Figure 2 and recorded their findings in the case record form (CRF).

#### Data collection.

The six COs performed 60 ultrasound studies each for a total of 360 studies on 168 patients. During the patients’ hospitalization, the pediatric patient underwent an ultrasound study with deidentified image stored and archived. For each patient scanned, 4-second lung ultrasound clips with two views in each zone (minimum 12 views per patient), which were labeled by location, was performed and recorded. After the CO completed the ultrasound study, they were instructed to immediately review the ultrasound image to record the diagnosis in the CRF. To maintain blinding, COs were instructed to not share their images or analyses with each other. Following the making of their diagnosis, COs were asked whether they had low, medium, or high confidence in their findings. The ultrasound images and CRF data files were uploaded to a secure server and were evaluated by two expert physician sonologists, with a third expert available in case of discordant results.

The first expert is a pediatric emergency medicine physician with a significant experience in pediatric lung ultrasound, whereas the second is a pediatric radiologist with ultrasound specialization. The third, tiebreaker, expert is an emergency medicine physician with an extensive global ultrasound experience. The expert physician sonologists were able to access the results of the ultrasound diagnostic data, with labeling of each zone (Figure 1), but were blinded to the COs’ CRF ultrasound interpretations, the COs’ reported confidence in the ultrasound findings, patients clinical diagnoses, and clinical information to serve as a reference standard,^14^ similar to the study by Shah et al.^16^ The first two experts scored every study, whereas the tiebreaker expert only evaluated discordant results. Similar to the study by Shah et al.,^16^ they scored the studies using the following two systems: American College of Emergency Physicians (ACEP) Quality Assurance 5-point Grading Scale (Table 1) and the binary questions. The two binary questions were as follows:

1.  “Are the images taken appropriate?” to signify that the image acquisition was sufficient for analysis.
2.  “Is the analysis acceptable?” to signify if it indicated the right ultrasound diagnosis.

**Table 1:** Quality assurance grading scale (from American College of Emergency Physicians emergency ultrasound standardized reporting guidelines 2011)^17^

1	No recognizable structures, no objective data can be gathered
2	Minimally recognizable structures, but insufficient for diagnosis
3	Minimal criteria met for diagnosis, recognizable structures but with some technical or other flaws
4	Minimal criteria met for diagnosis, all structures imaged well and diagnosis easily supported
5	Minimal criteria met for diagnosis, all structures imaged with excellent image quality and diagnosis completely supported

Discordant results between the two experts were defined as a difference in score of ≥ 2 on the ACEP Quality Assurance 5-point Grading Scale or any difference of opinion in the binary questions.

### Data entry and statistical analysis.

The patient information was entered into a specific online secure electronic database (REDCap, Vanderbilt University, Nashville, TN) by the designated data entry clerk. Patient information was collected using the paper CRF. This paper tool was then entered into the REDCap file, which was only accessible to individuals working on this project. The names of patients were not recorded in REDCap or on the ultrasound images.

Collected data included demographic factors, past medical history, physical examination findings, clinical and laboratory data, pediatric early warning scoring, vital statistics, and admission diagnosis. The COs recorded ultrasound diagnostic data such as location and type of abnormality, sonographic diagnosis, and confidence in the overall findings were collected.

We analyzed the data to show descriptive demographics of cases and patients, sonographic diagnosis by COs, reviewers’ assessment of the results, Cohen’s Kappa value for inter-rater agreement, and diagnostic test characteristics measured by sensitivity, specificity, and positive and negative likelihood ratios. For calculating Cohen’s κ, we compared COs’ sonographic diagnosis with whether the reviewers thought the analysis was appropriate.

Based on this approach, we calculated the number of those with and without the specific pathology (bronchiolitis, e.g.), stratified by test result. From this, we could calculate both the Cohen’s Kappa (κ) value and the diagnostic test characteristics such as sensitivity and specificity. We calculated CO sensitivity, specificity, likelihood ratios, and Cohen’s kappa using the reviewers’ and tiebreaker’s combined responses as a composite gold standard. We used Stata version 12 (Stata Corp, College Station, TX) for analysis.

## RESULTS

### Demographics and vital statistics.

The six ultrasound-naive COs cumulatively performed 360 ultrasound studies on 168 children. Demographic and clinical data on our study population are presented in Table 2.

**Table 2:** Demographic and vital statistics for children < 5 years old participating in study: Aweil, South Sudan

Category	Indicator (units)	Measure	Cases (*n* = 360)	Patients (*n* = 168)
Age	Months	Median [IQR]	12 [6–24]	10 [6–24]
Gender	Male	Number (%)	211 (59.3)	93 (56.4)
History and physical	Fever	Number (%)	220 (62.3)	104 (63.0)
	Difficulty breathing		304 (85.6)	142 (85.5)
	Chest indrawing		166 (47.9)	79 (49.4)
Admission vitals and laboratory*	Temperature (°C)	Median [IQR]	38.1 [36.6–38.8]	38.1 [36.9–38.9]
		Temp ≥ 38°C	203 (56.4)	103 (61.3)
	Respiratory rate (breaths/minute)	Median [IQR]	46 [40–56)	46 [40–56]
		Tachypnea (%)	184 (51.1)	85 (50.6)
	SpO2	Median [IQR]	98 [95–99)	98 [95–99]
		Number with SpO2 < 95% (%)	78 (21.7)	18 (11.0)
		Number with SpO2 < 92% (%)	40 (11.4)	19 (11.7)
	Heart rate (beats/minute)	Median [IQR]	154 [136–168)	154 [136–174.5]
		Tachycardia (%)	149 (41.4)	65 (38.7]
	Pediatric early warning scoring	Median [IQR]	4 [3–6)	4 [3–6]
	Rapid diagnostic test for malaria	Positive	62 (18.3)	36 (22.6)
		Negative	258 (76.3)	112 (70.4)
		Nothing Documented	18 (5.3)	11 (6.9)
	Blood sugar level (mg/dL)	Median [IQR]	112 [91–134]	112 [96–125]
	Hemoglobin (g/dL)	Median [IQR]	9.8 [8.5–11.3]	10.1 [8.5–11.6]

SPO2 = peripheral capillary oxygen saturation.

* Tachycardia was defined as a heart rate ≥ 190 beats/minute in children aged < 12 months and heart rate ≥ 140 beats/minute in children aged ≥ 12 months. Tachypnea was defined as a respiratory rate > 50 breaths/minute for children aged 2–11 months and a respiratory rate > 40 breaths/minute for children aged ≥ 12 months.

### Reviewers’ assessment.

A total of 355 ultrasounds studies of the 360 were reviewed by both expert reviewers as some studies were lost in transmission (five were lost in transmission to reviewer 1 and four were lost in transmission to reviewer 2). These two experts scored all the studies using two different methods as described earlier: 1) ACEP Quality Assurance 5-point Grading Scale and 2) binary yes/no grading of two questions “Are the images taken appropriate?” and “Is the analysis acceptable?”

On the ACEP Quality Assurance 5-point Grading Scale (1–5) questions, reviewers 1 and 2 gave a mean score of 3.87 and 4.57, respectively. Reviewers 1 and 2 disagreed on 29/352 (8.2%) of the studies by at least 2 points. These 29 studies were reviewed by the tiebreaker. The final average of the ACEP Quality Assurance 5-point Grading Scale, including the scores from reviewers 1 and 2 plus the tiebreaker review, was 4.11.

For the first binary question (“are the images taken appropriately?”), both reviewers 1 and 2 agreed that all 355 studies were appropriate. Subsequently, for the second binary question (“is the analysis acceptable?”), reviewers 1 and 2 found 82.8% (294/355) and 94.8% (336,356) of analyses acceptable, respectively, with disagreement on 64/355 cases (18.3%). The final average of the binary questions including scoring from reviewers 1 and 2 plus the tiebreaker found that for question 1, 99.1% of the images were appropriate, whereas for question 2, 86.0% of the analysis was acceptable.

Across the reviewers, each study took about 3.68 minutes to score.

Summing the discrepancies between the ACEP Quality Assurance 5-point Grading Scale (29 studies) and binary (65 studies) scoring systems and removing overlapping studies left 85 examinations for the tiebreaker reviewer to score (Table 3).

**Table 3:** Reviewers’ assessment of point-of-care ultrasound imaging and analysis by clinical officers: Aweil, South Sudan

Question	Measure	Reviewer 1 (355 studies)	Reviewer 2 (356 studies)	Average of scores (including 3rd reviewer when there was a discrepancy between reviewers 1 and 2)
ACEP Quality Assurance 5-point Grading Scale^17^	Mean	3.9 (3.8–3.9)	4.6 (4.5–4.6)	4.11
	1	0 (0)	0 (0)	0 (0)
	2	0 (0)	0 (0)	0%
	3	50 (14.2)	6 (1.7)	14%
	4	299 (84.9)	140 (39.3)	58%
	5	3 (0.9)	210 (58.9)	27%
Are the images taken appropriate?	Yes	355 (100)	356 (100)	99%
Is the analysis acceptable?	Yes	294 (82.8)	336 (94.4)	86%
	No	61 (17.2)	20 (5.6)	14%
Time to review study	Median [IQR]	3 [3–3]	5 [4–5]	–

### Diagnostic test characteristics.

Cohen’s κ for more specific conditions shows results of 0.81 (95% CI: 0.74–0.87) for viral pneumonia, 0.73 (95% CI: 0.63–0.82) for bacterial pneumonia, and 0.49 (95% CI: 0.32–0.67) for interstitial syndrome. Clinical officer test performance characteristics are presented in Table 4 using a composite of expert reviewers with tiebreaker as a reference gold standard.

**Table 4:** POCUS diagnostic test characteristics in Aweil state hospital, South Sudan

	Sensitivity	Specificity	LR+	LR−	Cohen’s κ
Lung consolidation/bacterial pneumonia	69 (58–78)	98 (95–99)	30.5 (12.6–73.4)	0.3 (0.2–0.4)	0.7 (0.6–0.8)
Bronchiolitis or viral pneumonia	85 (78–90)	96 (91–98)	21.2 (9.6–46.1)	0.2 (0.1–0.2)	0.8 (0.7–0.9)
Interstitial Syndrome*	40 (25–55)	99 (97–100)	33.6 (10.3–109.8)	0.6 (0.5–0.8)	0.5 (0.4–0.6)

LR+ likelihood ratio for a positive test; LR− likelihood ratio for a negative test.

* Interstitial syndrome referring to acute respiratory distress syndrome or severe viral lower respiratory tract infection, less commonly pulmonary edema.^15^

### Diagnosis of distribution by COs.

Among the 360 cases, 88 (24.4%) were considered to be normal. The most common abnormal finding using POCUS was bronchiolitis or viral pneumonia (51.4%), followed by bacterial pneumonia (30.0%) and interstitial syndrome (9.7%). The disease profile was largely similar between cases and unique patient sub-cohort (Table 5). The proportion of patients with bronchiolitis and consolidation was slightly higher among patients with an initial admission diagnosis of pneumonia or tuberculosis. The COs reported low confidence for 0% of studies, medium confidence for 30.6% of studies (*n* = 110), and high confidence for 56.7% of studies (*n* = 204). The median time to perform the ultrasound study, including pre-discussion with the caretaker, was 15 minutes (IQR 12–22 minutes).

**Table 5:** Sonographic diagnosis by ultrasound imaging by clinical officers

Ultrasound impression	All cases (*n* = 360)	Unique patients (*n* = 168)	Among cases with diagnosis of pneumonia (*n* = 220)	Among cases with diagnosis of TB (n = 22)
Normal	88 (24.4)	48 (28.6)	48 (21.3)	2 (9.1)
Bronchiolitis or viral pneumonia	185 (51.4)	82 (48.8)	121 (53.8)	13 (59.1)
Consolidation/bacterial pneumonia	108 (30.0)	49 (29.2)	82 (36.4)	11 (50.0)
Interstitial syndrome*	35 (9.7)	13 (7.7)	11 (4.9)	2 (9.1)
Pleural effusion	0 (0.6)	2 (1.2)	2 (0.9)	1 (4.6)
Pneumothorax	0 (0)	0 (0)	0 (0)	0 (0)
Other	0 (0)	0 (0)	0 (0)	0 (0)

* Interstitial syndrome referring to acute respiratory distress syndrome or severe viral lower respiratory tract infection, less commonly pulmonary edema.^15^

## DISCUSSION

Lung POCUS has previously been shown to have a high diagnostic accuracy to diagnose pneumonias^9^ and has recently shown to be useful in diverse settings.^11^ Similar to the prior literature from multiple settings,^9–11,16,18,19^ we report high specificities (Table 4) to rule in respiratory pathologies, with variable sensitivities to rule out diseases for lung ultrasound performed by novice COs.^12^ There was high interobserver agreement as measured by Cohen’s kappa for bacterial pneumonia and bronchiolitis/viral LRTI consistent with prior studies.^14,18,20^ Being able to expand this imaging modality to settings with limited diagnostic capacities (such as X-rays) can advance care for children with LRTIs. In addition, these results are significant as they may assist in improving antibiotic stewardship.^4^ This study demonstrates that learning a POCUS algorithm to distinguish LRTI and other causes of respiratory distress in children < 5 years of age after a 12-hour training program in low-resource settings is feasible.

Our study evaluated the ability of COs in South Sudan to learn a lung POCUS algorithm, as graded by expert reviewers using two different scoring systems. In the first scoring system, the average score on the 5-point ACEP quality assurance grading scale^17^ was 4.1, which is high. The second scoring system shows that COs could acquire adequate lung ultrasound images at a very high level (99.1%) and analyze these images at appropriately 86.0% (Table 3). Overall, these initial results show a high quality of imaging acquisition skill and a good image analysis skill among the COs. The specificity of CO who performed lung ultrasound to rule in pneumonia was very high, with lower sensitivity to rule out pneumonia consistent with other data using novice sonologists (ultrasound-naive medical student, and pediatric emergency fellow in Lissaman et al.^8^: sensitivity 71% and specificity 85%; and novice pediatric resident in Zhan et al.^7^: sensitivity 40% and specificity 91%). This study demonstrates strong potential for use of POCUS algorithms in South Sudan by mid-level providers, which should iteratively improve with further individual practice and improvements in training methodologies. These improvements include focusing on predictable errors, more case-based presentations during didactic sessions, group-based image review to have collaborative experience, peer-to-peer coaching, and self-paced image review tools.

Secondary, analysis of more specific disease-specific capacity showed that they were better at detecting bronchiolitis/viral pneumonia and bacterial pneumonia than interstitial syndrome. The interobserver agreement (Cohen’s κ) between the combined the expert reviewers and the individual CO analysis was very good for bronchiolitis/viral pneumonia (Cohen’s κ = 0.8), good for bacterial pneumonia (Cohen’s κ = 0.7), but only moderate for interstitial syndrome (Cohen’s κ = 0.5) (Table 4). This lower score for interstitial syndrome may have been a teaching deficiency with confusion about what constitutes a positive region of B-lines and how many minimal regions/zones are needed to constitute interstitial syndrome. As per international consensus, a positive region/zone is defined “by the presence of three or more B-lines in a longitudinal plane between two ribs.”^15^ Different criteria for the number of regions/zones are used. In this study using a six-zone protocol, a minimum of five positive zones with three or more B-lines or confluent B-lines were required for interstitial syndrome. However, this may not have been clearly defined in the teaching tools. This reflects the need to ensure straightforward algorithms for nonexpert users of POCUS.

Further analysis of disease-specific test performance characteristics of lung POCUS shows sensitivity and specificity for viral LRTI were ≥ 85% and comparable to results from previous studies,^9,14^ whereas for bacterial pneumonia, sensitivity was lower and specificity higher than those of prior studies.^18^ Overall, these differences did not constitute any significant discrepancy in the efficacy of POCUS in the contexts of South Sudan relative to countries such as the United States.^7,10^ Ultimately, the diagnostic test characteristics suggest that POCUS could be of high utility in the low-resource clinical settings.

This study adds significant findings to the literature on pediatric LRTIs. To our knowledge, this is one of the first studies to investigate the feasibility of using POCUS for diagnosing LTRIs among children younger than five years in South Sudan. Also, in contrast to many other prior studies of POCUS teaching in other countries, our sonologists (clinicians who perform and interpret ultrasound) were COs and not physicians. This is particularly relevant for resource-limited, rural, and conflict-ridden settings often with a shortage of physicians where mortality due to LRTIs may be high.^1^ Moreover, having expert reviewers who have extensively used POCUS technology previously also helped assess the validity of the analyses accurately. This is an especially important consideration where usual standard diagnostic tests such as chest X-rays are frequently not available. Finally, the use of a 12-hour standardized teaching curriculum clearly defines the intervention and clarifies the specific effect of the training program on COs’ ability to diagnose LRTI using POCUS. Rather than a general medical training or radiology, such a targeted training program can exhibit quicker and more dramatic improvements in outcomes after relatively small investment in operations research. Further investment can be made to address additional fundamental questions, including proper antibiotic choices and stewardship.

Additional research is also needed on how to improve the existing MSF training methodology. Although prior studies have demonstrated the feasibility of conducting such examinations in 7 minutes (image acquisition only),^18^ the median time it took to conduct a POCUS examination was (including setup, explanation to parents, and written informed consent) 15 minutes in Aweil. Determining how to conduct the examination quickly but with higher accuracy will be essential for making lung ultrasound easier to integrate into busy, under-resourced hospitals in LMICs. Further research could also show how to interpret lung POCUS findings in contexts with high prevalence of other diseases affecting pulmonary status, including malaria, tuberculosis, and pediatric cardiac diseases, including congenital and rheumatic heart disease. One promising method would be to combine a basic lung and cardiac ultrasound into a syndromic “dyspnea” algorithm, which has the potential to elucidate the primary cause or causes of respiratory distress through bedside ultrasound.^16^ This further research could potentially impact health outcomes such as length of stay, readmission rates, related complication rates, and mortality from LRTI. Finally, understanding the financial and logistic implications of implementing a POCUS program is necessary to properly balance the utilization of resources and scale-up POCUS in similar settings. These implications are presently being evaluated through additional POCUS trainings in Aweil, as part of an integrated implementation of diverse POCUS uses by COs.^16^

### Limitations.

Our study may be limited by the lack of a traditional reference standard such as chest X-ray, which has previously been used as a standard comparator for diagnosing pneumonia in prior work. However, chest X-ray as a reference gold standard was not feasible in our resource-limited setting.^9,16,21^ A systematic review and several studies have demonstrated lung ultrasound to be as accurate as chest X-ray in multiple settings with very high inter-observer agreement as measured by Cohen’s kappa.^9–11,14,16,18,21^ Furthermore, we attempted to mitigate this limitation by having multiple blinded expert reviewers who assessed and reviewed all ultrasound images acquired and interpreted by COs similar to a study in a resource-limited setting by Shah et al.^16^

## CONCLUSION

It is feasible to train COs in South Sudan to use a POCUS algorithm to diagnose pneumonia and other pulmonary diseases in children < 5 years old through a focused, field-based training. The results of this study can be used by clinicians and policymakers to assess and design policies that address the unmet diagnostic needs of LRTIs in pediatric populations and reduce their associated morbidities and mortalities in low-resource settings.

## Supplemental appendix

Supplemental materials
