# Supplementary material for: Feasibility of Training Clinical Officers in Point-of-Care Ultrasound for Pediatric Respiratory Diseases in Aweil, South Sudan
Source: Am J Trop Med Hyg. 2019 Jul 8;101(3):689–95. doi: 10.4269/ajtmh.18-0745 (PMC6726960; doi:10.4269/ajtmh.18-0745)
Supplement: Supplementary file 1 [file tpmd180745.SD1.pdf]

Supplemental Appendix – Training Methodology

General information

- 2 participants per day, 2 days each participant

Day	1	2	3	4	5	6
Participant	1 & 2	3 & 4	5 & 6	1 & 2	3 & 4	5 & 6

- Schedule each day – 6 hours/day
    - 8AM-9AM – didactic using powerpoints
    - 11AM-1PM – bedside scanning
    - 1PM-2PM lunch
    - 2PM-3PM – image review from AM and didactic review
    - 3PM-5PM – bedscide scanning and image review at end
